# Chemical Composition and Bioactive Antioxidants Obtained by Microwave-Assisted Extraction of *Cyperus esculentus* L. By-products: A Valorization Approach

**DOI:** 10.3389/fnut.2022.944830

**Published:** 2022-07-08

**Authors:** Carlos Javier Pelegrín, Marina Ramos, Alfonso Jiménez, María Carmen Garrigós

**Affiliations:** Department of Analytical Chemistry, Nutrition and Food Sciences, University of Alicante, San Vicente del Raspeig, Spain

**Keywords:** *Cyperus esculentus* L., chemical composition, antioxidants, microwave-assisted extraction, optimization, valorization, *horchata* industry

## Abstract

Tiger nut is highly appreciated in the Mediterranean basin by the large number of nutritional advantages offered by a beverage, called “*horchata*,” which is directly obtained from the tuber of *Cyperus esculentus* L. However, the current tiger nut harvesting and processing practices generate a large number of residues, mainly a solid by-product after processing and the plant that remains spread out in the fields. In this work the plant residues have been fully characterized to get a clear picture of the possibilities for its valorization to generate products with high added value. Several analytical techniques have been applied to obtain data to assess the real possibilities of these residues in advanced applications in the food, packaging and nutrition sectors. Results on the compositional and elemental analysis, monosaccharide composition, phenolic concentration, and antioxidant capacity were obtained from the dry powder (DP). The high content of α-cellulose (47.2 ± 1.8%) in DP could open new possibilities for these residues as raw material in the production of cellulose nanoentities. Many essential minerals with nutritional interest (Na, Mg, Ca, Mn, Fe, Cu, and Zn) and free sugars (xylose, arabinose, glucose, and galacturonic acid) were identified in the DP making it an interesting source of valuable nutrients. The total carbohydrate content was 171 ± 31 mg g_dm_^–1^. In addition, microwave-assisted extraction (MAE) was used to obtain extracts rich in polyphenolic compounds. A Box–Behnken design (BBD) was used, and the optimal extraction conditions predicted by the model were 80°C, 18 min, ethanol concentration 40% (v/v), and solvent volume 77 mL, showing an extraction yield of 2.27 ± 0.09%, TPC value was 136 ± 3 mg_*GAE*_ 100 g_dm_^–1^ and antioxidant capacity by the ABTS method was 8.41 ± 0.09 μmol_*t*rolox_ g_dm_^–1^. Other assays (FRAP and DPPH) were also tested, confirming the high antioxidant capacity of DP extracts. Some polyphenols were identified and quantified: *p*-coumaric (7.67 ± 0.16 mg 100 g_dm_^–1^), ferulic (4.07 ± 0.01 mg 100 g_dm_^–1^), sinapinic (0.50 ± 0.01 mg 100 g_dm_^–1^) and cinnamic acids (1.10 ± 0.03 mg 100 g_dm_^–1^), 4-hydroxybenzaldehyde (1.28 ± 0.06 mg 100 g_dm_^–1^), luteolin (1.03 ± 0.01 mg 100 g_dm_^–1^), and naringenin (0.60 ± 0.01 mg 100 g_dm_^–1^). It can be concluded that *C. esculentus* L. residues obtained from the tiger nut harvesting and *horchata* processing could be an important source of high value compounds with potential uses in different industrial sectors, while limiting the environmental hazards associated with the current agricultural practices.

## Introduction

Tiger nut or *chufa* is a tuber obtained from the perennial plant *Cyperus esculentus* L. var. *sativus* Boeck., traditionally cultivated in Southern Europe, Africa, and the Middle East, which has been cultivated since ancient times by its culinary and medicinal properties (1). Tiger nut cultivation is significant in the Mediterranean area, and it is used as a snack and mainly as raw material for a sweet and tasty beverage called “*horchata*,” which is considered highly valuable from the nutritional point of view (2). The tiger nut plant (*C. esculentus* L.) is a herb around 40–50 cm high, with roots formed by many rootlets where tiger nuts can grow in elongated or rounded forms. Most of the tiger nut tubers have elongated shape, although they can also have a round appearance with 8–16 mm diameter (3). It has been stated that tiger nuts offer significant human health benefits, such as controlling cholesterol and triglycerides, vasodilating effects, and antidiarrheal properties (4). These tubers are rich in essential dietary constituents, such as fibers, minerals, fatty acids, carbohydrates, proteins, and vitamins (5, 6).

This crop is characteristic of a specific area near Valencia (Spain), by the ideal soil (well-drained sandy or loamy soils with pH 5.0 to 7.5) and climatic conditions (temperatures alternating between day and night in the range 20–30°C) for tiger nut growing, resulting in the protection by the designation of origin “*Chufa de Valencia*.” Around 8,360 tons of dried tiger nuts are produced annually in this area, representing their main business sector, particularly during the cultivation period between April and November (1, 7). In Spain, tiger nut is cultivated in rotation with other vegetables such as onion, potato, watermelon, carrot, cabbage, and artichoke (8). However, the production of tiger nut results in a large amount of harvesting residues, since the perennial plant must be wholly withered, and sun dried to obtain the tuber in a process that can take up between 1 and 3 months. Subsequently, the aerial part of the plant can be considered a residue and it is currently removed by controlled burning, generating greenhouse gases and ashes that remain in the soil and contribute to its impoverishment (9).

**FIGURE 1:**
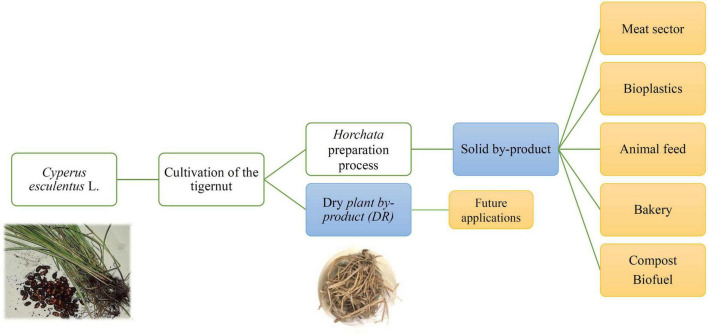
Scheme of by-products generated during *horchata* preparation.

On the other hand, tubers are used to produce *horchata*, but a major fraction of the raw material results in a solid residue, which should also be removed during processing. It has been reported that the average liquid/solid *horchata* production yield is around 53 wt.% for the liquid and 47 wt.% for the solid residue (5), but more recent data showed some increase in the liquid production yield up to 70 wt.% (10). Besides, the waste management during tiger nut harvesting and milk extraction represents a significant problem, with some economic and environmental issues to be urgently evaluated, as shown in Figure 1. For all these reasons, farmers and producers require the proposal of alternative uses for these wastes since the current applications are composting, organic matter, or animal feed, all of them representing low-added value uses (8, 11, 12). These solid residues have also been used as co-products by the food industry, in particular, to increase the fiber content in meat or pastry (13–15), as well as in the manufacture of biocomposites with high fiber content (16).

Recent studies have focused on the chemical properties of tiger nut based on its composition (proteins, ash, moisture, fiber, fat, and carbohydrates), minerals, total polyphenols, antioxidant, and antibacterial content (17–20). On this basis, some innovative applications of tiger nut residues have been recently proposed. For instance, Kani et al. (21, 22) reported the potential of these residues as a green and sustainable adsorbent of colorants in different solutions, but this application is too specific to get a significant economic income for farmers and producers. Tiger nut residues have also been considered a valuable source of bioactive antioxidant compounds and fatty acids. Different strategies for extracting these compounds from these by-products have been reported. Roselló-Soto et al. (23, 24) proposed the use of conventional solid-liquid (Soxhlet) as well as innovative supercritical carbon dioxide (SC-CO_2_) extraction methods to get monounsaturated fatty acids, phytosterols, proteins, and other bioactive compounds. Other authors proposed using gas-assisted mechanical expression (GAME) as a promising technology for the recovery and purification of oil and polyphenols from tiger nuts (25–27). These strategies resulted in the successful extraction of palmitic and linoleic acid, vitamin E (α-tocopherol and β-tocopherol), β-sitosterol, or esterified phenolic acids such as ferulic acid and *p*-coumaric acid. Finally, ultrasound-assisted extraction (UAE) has been used by other authors to modify proteins previously extracted from tiger nuts to improve and promote their use by the food industry (28). All these compounds were obtained from the solid by-products of the “*horchata*” production.

Nevertheless, the available information about the valorization of the *C. esculentus* L. plant as a source of bioactive compounds is scarce, and further studies are necessary to get the complete picture of the possibilities offered by this agricultural residue. Therefore, the study of its elemental composition has been carried out in this study to assess the potential as nutrients source of this agricultural by-product, while also limiting the environmental problems associated with the current burning practices after harvesting. Data about the complete composition (extractives, fiber, fats, proteins, minerals, carbohydrates, and bioactive compounds with potential antioxidant capacity) of this by-product are necessary to evaluate the possibilities of obtaining cellulose nanocrystals and other interesting bio-based materials, considering the background obtained from similar plants, such as flax fibers, rice-straw, or sugarcane bagasse (29–31). Jing et al. (32) reported the potential of *C. esculentus* L. leaves to obtain flavonoids by using dynamic high-pressure microfluidization (DHPM). These authors concluded that the antioxidant capacity and the bacteriostatic performance against Gram-positive and Gram-negative bacteria of these flavonoids were high, and they could be successfully used by the food and pharmaceutical industry. Other advanced extraction technologies, including microwave-assisted extraction (MAE) and UAE, supercritical fluid extraction and pulsed electric field extraction, have shown their potential to get pure fractions of bioactive compounds from different vegetable materials by maximizing their yields in short extraction times with less amount of solvent and lower costs for the overall process (33).

In this work, the complete characterization of the *C. esculentus* L. plant was carried out for the first time. In addition, the optimal MAE conditions for extracting polyphenols from the plant were determined by using the response surface methodology (RSM). The antioxidant capacity of these extracts was also determined while the main polyphenols were identified and quantified by ultra-high-performance liquid chromatography-electrospray ionization-tandem mass spectrometry (UHPLC-ESI-MS/MS). The information provided in this study will be helpful for nutritionists, consumers, farmers, and entrepreneurs in the food industry for the further valorization of the harvesting residues obtained during the *horchata* processing, while also helping to solve a significant environmental problem in those areas where tiger nut is cultivated.

## Materials and Methods

### Materials and Sample Preparation

Cultivated *C. esculentus* L. plants were kindly supplied by Terra i Xufa (Foios, Valencia, Spain) once harvested and prior to their incineration. Plants were cleaned with water to eliminate mud and weeds and dried in an oven at 40°C for 24 h. Dry samples were ground and sieved in a Retsch ZM 200 ultracentrifugal mill (Haan, Germany) with a 0.4 mm pore mesh. The obtained dry powder (DP) was stored in the dark for subsequent analysis under vacuum at room temperature.

TPTZ [2,4,6-Tris(2-pyridyl)-s-triazine, ≥98%]; gallic acid monohydrate (≥99%); trolox (6-hydroxy-2,5,7,8-tetramethylchroman-2-carboxylic acid, 97%); DPPH radical (2,2-diphenyl-1-picrylhydrazyl, 99%); glucose; fucose; galactose; arabinose; xylose; mannose; ribose; fructose; galacturonic acid; and glucuronic acid were supplied by Sigma-Aldrich (Madrid, Spain). All other chemicals and reagents (analytical grade) were purchased from Sigma-Aldrich (Madrid, Spain).

### Proximate Composition Analysis

Proximate composition of the tiger nut plant (moisture, fat, ash, proteins, and carbohydrates) was analyzed, in triplicate, according to the AOAC methods (34). Briefly, moisture content was determined after drying samples at 105 ± 5°C until constant weight. Crude protein content was evaluated by using an automatic macro-Kjeldahl distillation and titration unit (Pro-Nitro-M, JP Selecta, Barcelona) (AOAC 978.04) by measuring the nitrogen content (N × 6.25). Ash content was determined by incineration in a muffle furnace at 600 ± 15°C (AOAC 923.03), and crude fat content was obtained by Soxhlet extraction with petroleum ether for 8 h (AOAC 920.85). Total carbohydrates were determined by difference according to Eq. 1:


$$Totalcarbohydrates\left(\frac{g}{100}g_{f\,r\,e\,s\,h\,m\,a\,t\,e\,r\,i\,a\,l}\right)=100-(g_{m\,o\,i\,s\,t\,u\,r\,e}$$

(1) $$+g_{f\,a\,t}+g_{a\,s\,h}+g_{p\,r\,o\,t\,e\,i\,n\,s})$$

TAPPI Standards were used to evaluate water solubility (TAPPI T207 cm-99) and 1% sodium hydroxide solubility (TAPPI T212 om-02). Finally, the extractives content (TAPPI T204 cm-97), acid-insoluble lignin (TAPPI T222 om-02), holocellulose (35) and α-cellulose and hemicellulose contents were determined (36). The hemicellulose content was calculated by subtracting the α-cellulose weight from the holocellulose amount.

### Mineral Analysis

Approximately 0.2 g of DP were digested in a closed vessel with 8 mL of 6:2 HNO_3_ (65%, v/v)/H_2_O_2_ (30%, v/v) using a microwave digestion system (MLS-ETHOS plus, Milestone srl, Bergamo, Italy). Digestion conditions were as follows: 15 min to reach 200°C, maintaining this temperature for 15 min and cooling for 30 min. The digested sample was made up to 50 mL with Milli-Q-ultrapure water, and filtered through a 0.22 μm nylon membrane. The mineral composition of DP was determined, in triplicate, using inductively coupled plasma optical emission spectrometry (ICP-OES), using an Optima 7300 DV instrument (PerkinElmer, Inc., Shelton, CT, United States). Operating conditions were: 1,300 W radiofrequency power, 15 L min^–1^ plasma flow, 2.0 L min^–1^ auxiliary flow, 0.8 L min^–1^ nebulizer flow, and 1.5 mL min^–1^ sample uptake rate. Calibration standards were prepared from a multi-element standard solution of 1,000 mg L^–1^ (23 elements in diluted nitric acid, Merck, Darmstadt, Germany) after appropriate dilutions.

### Monosaccharide Composition

The monosaccharide composition of DP was determined by following the procedure proposed by Shi et al based on trifluoroacetic acid (TFA) hydrolysis and two-step sulfuric acid hydrolysis processes (37). In brief, sample (2 mg) was incubated with 125 μL of 72% H_2_SO_4_ at room temperature for 3 h. Then, the solution was diluted with 1.375 mL of deionized water and incubated at 100°C for 3 h. For the TFA hydrolysis, the same amount of sample (2 mg) was incubated with 1 mL of TFA (2 M) at 120°C for 3 h, cooled down and dried under an air stream before dissolution with ultrapure water. The monosaccharides were analyzed by using high performance anion exchange chromatography with pulsed amperometric detection (HPAEC-PAD) with a 940 IC system (Metrohm, Madrid, Spain) equipped with a Matrosep Carb 2 column (4 × 250 mm, Metrohm). Inositol was added as internal standard prior to hydrolysis. All experiments were carried out in triplicate. Results were expressed in mg per g of dry weight.

### Microwave-Assisted Extraction of Phenolic Compounds

Microwave-assisted extraction was performed using a flexiWAVE microwave equipment (Milestone srl, Bergamo, Italy). Aqueous ethanol solutions were used as a safe and efficient solvent for extracting phenolic compounds (33). A total of 3 g of DP were introduced into a round bottom flask and magnetically stirred. The studied MAE parameters were temperature, ethanol concentration, extraction time, and solvent volume. After MAE, DP extracts were centrifuged at 5,300 rpm for 10 min to separate the solid residue. The supernatant was frozen at −20°C for 24 h to precipitate possible interfering compounds and centrifuged again under the same conditions. Then, the ethanol present in the supernatant was evaporated at 55°C using a vacuum rotary evaporator (R II, Buchi Labortechnik AG, Flawil, Switzerland). Finally, extracts were freeze-dried and reconstituted with ethanol 40%, v/v, immediately before analysis.

Microwave-assisted extraction optimization of bioactive compounds was carried out by using a Box–Behnken design (BBD) with four independent variables (temperature, 40–80°C; ethanol concentration, 40–80%, v/v; extraction time, 5–40 min; and solvent volume, 50–80 mL) at three levels. This design consisted of 29 experiments, including five central points. All experiments were performed randomly, and the range of the studied variables was selected according to preliminary tests and experimental limitations. The responses obtained were evaluated regarding extraction yield, total phenolic content (TPC) and antioxidant activity (ABTS assay). RSM was used to determine the optimal extraction conditions. The regression analysis of experimental data was performed according to Eq. 2 for obtaining the quadratic polynomial empirical models:


(2) $$\text{Y}=\beta_{0}+\Sigma \,\beta_{i}\,X_{i}+\Sigma \,\beta_{\mathrm{ii}}\,X_{i}^{2}+\Sigma \,\Sigma \,\beta_{\mathrm{ij}}\,X_{i}\,X_{j}$$

where *Y* is the predicted response; *X* represents the variables of the system; *i* and *j* are independent variables; β_0_ is the intercept coefficient; β_*i*_ is the linear coefficient; β_ii_ is the quadratic coefficient; and β_ij_ is the interaction coefficient of *i* and *j* variables.

The prediction of the optimal parameters for polyphenols extraction was performed based on regression and response surface plots analysis. A verification test under optimal extraction conditions was carried out by comparing experimental results, in triplicate, with predicted values to confirm the model validity.

### Dry Powder Extract Characterization

#### Extraction Yield

The extraction yield was calculated according to Eq. 3:


(3) Extractionyield(%)=W1W0x 100

where *W*_1_ and *W*_0_ are the weights of the final dry extract and the initial sample, respectively.

#### Total Phenolics Content

Total phenolics content was determined, in triplicate, by using the Folin–Ciocalteu colorimetric assay according to a previously described method with some modifications (38). Briefly, 100 μL of the DP extract was mixed with 750 μL of a 10-fold diluted Folin–Ciocalteu reagent. Solutions were mixed thoroughly and incubated at room temperature for 5 min. Then, 750 μL of a 7.5% sodium carbonate solution was added, and the mixture was incubated in the darkness at room temperature for 90 min. The absorbance was determined at 725 nm using a Biomate 3 UV-vis spectrophotometer (Thermospectronic, Mobile, AL, United States). Results were expressed as mg of gallic acid equivalents (GAE) per 100 g of dry weight. All samples were analyzed in triplicate.

#### Phenolics Profile by Ultra-High-Performance Liquid Chromatography-Electrospray Ionization-Tandem Mass Spectrometry Analysis

The identification and quantification of phenolic compounds in DP were performed by UHPLC coupled to a triple quadrupole spectrometer (UHPLC-1290/QqQ-6490, Agilent Technologies, Palo Alto, CA, United States). The instrument was equipped with a vacuum degasser, a binary pump, and a thermostated autosampler and column compartment. Compounds were separated with a BRISA LC2 C18 column (150 mm × 4.6 mm × 5 μm, Teknokroma, Barcelona, Spain). Water (A) and acetonitrile (B) were used as solvents in the mobile phase, both containing 0.1% (v/v) formic acid. The elution gradient was carried out at a constant flow rate of 0.5 mL min^–1^, as follows: 0 min, 90% A; 25 min, 60% A; returning to initial conditions at 29 min. The injection volume was 10 μL. The autosampler and column were kept at room temperature. Mass spectra were recorded in the m/z range of 50–800. The electrospray chamber was set at 3.5 kV with a desiccant gas temperature of 275°C. The pressure and flow rate of N_2_ in the nebulizer were 40 psi and 11 L min^–1^, respectively, and the MS/MS collision energies were set at 10, 20, 30, and 40 V.

The UHPLC system was coupled to the mass analyzer with an electrospray ionization interface (ESI) operating in the negative ionization mode, showing [M−H]^–^ molecular ions for *p*-coumaric acid; and in the positive ionization mode, showing [M−H]^+^ molecular ions for ferulic acid, 4-hydroxybenzaldehyde, naringenin, sinapinic acid, and cinnamic acid. The phenolic compounds were identified by comparing their fragmentation pattern and retention times with those of standard solutions of all these compounds. Quantification was performed using the multiple reaction monitoring (MRM) technique once the characteristic fragments of each compound were analyzed and calibration curves of the target phenolic standards were obtained. Results were expressed in mg polyphenol 100 g^–1^ of DP.

#### Antioxidant Activity

The antioxidant activity against 2,2′-azino-bis(3-ethylbenzothiazoline-6-sulfonic acid) (ABTS) was determined, in triplicate, according to Masci et al. (39) with slight modifications. ABTS radical cations were prepared by mixing 5 mL of 7 mM ABTS solution with 88 μL of 140 mM potassium persulfate solution. The obtained solution was kept in the dark for 12–16 h. Then, the ABTS stock solution was conveniently diluted with ethanol until obtaining an absorbance value of 0.80 at 734 nm. Afterwards, 100 μL of the extract were mixed with 2 mL of the adjusted ABTS solution and incubated at room temperature for 30 min. Finally, the absorbance was measured at 734 nm against a pure ethanol blank. Results were expressed as μmol of trolox equivalents (TE) per g of dry weight.

The scavenging activity of DPPH free radicals was determined, in triplicate, by using a previously described method with some modifications (40). Briefly, 2.7 mL of DPPH solution (6 × 10^–5^ M in ethanol) were added to 0.3 mL of different concentrations of sample solutions in 60% (v/v) ethanol. Mixtures were shaken vigorously and incubated in the dark for 30 min at 37°C. Then, the absorbance was measured at 517 nm using ethanol as control. Results were expressed as EC_50_ (concentration of sample needed to scavenge 50% of the DPPH radicals) and calculated based on a calibration curve obtained by plotting the concentration of sample solutions and the corresponding scavenging effect.

The ferric ion reducing antioxidant power (FRAP) was also determined, in triplicate, as previously described by Moure et al. (41), with slight modifications. The FRAP reagent was freshly prepared by mixing acetate buffer (300 mM, pH = 3.6), TPTZ (10 mM), and ferric chloride (20 mM) at a 10:1:1 (v/v/v) ratio, respectively. Then, 3 mL of the FRAP reagent and 100 μL of the sample solution were mixed and incubated at 30°C for 30 min. The absorbance was then determined at 593 nm using a UV-vis spectrophotometer. Results were expressed as μmol of TE per g of dry weight.

### Statistical Analysis

Statistical analysis of results was performed using STATGRAPHICS Centurion XVI (Statistical Graphics, Rockville, MD, United States). A one-way analysis of variance (ANOVA) statistical comparison was performed using the Tukey test to discriminate between means at a 95% confidence level. Graphic analysis of the main effects and interactions between variables was used to determine the optimal extraction conditions. A least-squares regression analysis was performed to determine the coefficients of the polynomial quadratic models. Lack of fit, *F* test and determination coefficients (*R*^2^) were obtained from ANOVA to evaluate the adequacy of the fitted models at a confidence level of 95%.

## Results and Discussion

### Chemical Composition of Dry Powder

The main parameters of the chemical composition of DP are reported in Table 1. A high ash content (6.9 ± 0.2%) was obtained in the plant-derived residue, indicating a high content of inorganic elements with nutritional interest. The moisture content of DP was 5.4 ± 0.1%, while protein and fat contents were 1.6 ± 0.2 and 1.1 ± 0.2%, respectively. Just a limited number of articles are available in the literature dealing with the chemical composition of the *C. esculentus* L. plant, with some of them focused on the edible tuber (42, 43) or related to other *Cyperus* plants (44), such as *Cyperus rotundus* (45), *Cyperus articulatus* (46), or *Cyperus papyrus* (47). Considering the chemical composition of similar plants, it can be stated that results reported for *Cissus populnea* stems (48) showed higher values for all the studied parameters than those obtained in this study; while higher protein content (6.1 ± 0.4%) and lower fat content (0.002%) were reported for *Cyperus tegetum* (49).

**TABLE 1:** Chemical composition of DP (*n* = 3, mean ± SD).

Composition	Dry plant by-product (DP) (%)
Moisture	5.4 ± 0.1
Ash	6.9 ± 0.2
Protein	1.6 ± 0.2
Fat	1.1 ± 0.2
Carbohydrates	84.9 ± 0.5
Solvent extractives	3.9 ± 0.3
Klason lignin	29.3 ± 0.7
Holocellulose	60.0 ± 1.2
α-Cellulose	47.2 ± 1.8
Hemicellulose	13.0 ± 2.0
H_2_O solubility	37.1 ± 0.6
NaOH 1% solubility	14.9 ± 0.3

*Results are expressed on a dry matter basis.*

The carbohydrate content of DP was 84.9 ± 0.5%, being significantly higher than values reported for *Cyperus capitatus* and *Cyperus conglomeratus*, which ranged from 50 to 57% (50). According to these authors, the dry matter of forage crops should contain about 50–80% carbohydrates, in line with the results obtained for DP. Other major components present in DP were holocellulose (60.0 ± 1.2%), followed by lignin (29.3 ± 0.7%), and extractives (3.9 ± 0.3%). These results agree with those reported in several studies for similar plants such as rice, wheat, and barley straw (51), finding 70–80% of holocellulose, around 20% of lignin, and 1–4% of extractives. In addition, DP proved to be a rich source of α-cellulose (47.2 ± 1.8%), showing higher content than other agricultural by-products such as rice (36.9 ± 1.4%), wheat (43.0 ± 2.3%), and barley straw (39.2 ± 2.6%) (49, 51). As a result, this by-product could be a potential source of cellulose to obtain cellulose nanocrystals. The Klason lignin content of DP was comparable to the value obtained by Rosado et al. (47) from the rind of the papyrus (*C. papyrus L.*) plant, accounting for 27.0% of the dry material, while lignin content in the pith was significantly lower, accounting for only 15.7% of the dry material.

Dry Powder solubility in H_2_O and NaOH 1% showed values of 37.1 ± 0.6 and 14.9 ± 0.3%, respectively. Mondragon et al. (52) studied similar plants and residues, such as sisal, hemp and flax fibers, with values ranging between 14.2 ± 0.1 and 20.7 ± 0.4% for NaOH 1% solubility and between 3.2 ± 0.3 and 6.9 ± 0.1% for H_2_O solubility. These differences in chemical composition may be due to the different geographical origin, cultivar, maturity and sample preparation (including drying, extraction, and analysis) (47). Besides, the chemical composition of DP demonstrated that this residue has the potential to be used as a source of carbohydrates, especially α-cellulose, which is a valuable material in several sectors, such as cosmetics or packaging.

### Mineral Composition

The elemental analysis of DP was carried out and results are shown in Table 2. Many dietary essential minerals which develop key functions necessary for our organism and health, such as Na, Mg, Ca, Mn, Fe, Cu, and Zn were identified (53). Good levels of linearity were obtained for all calibration curves with determination coefficients (*R*^2^) higher than 0.999 for all the studied elements, at six calibration points (concentration range 0.1–50.0 mg kg^–1^) run in triplicate. Values for the limit of detection (LOD) and limit of quantification (LOQ) were calculated from the regression parameters of the calibration curves as *3 S_*y/x*_/a* and *10 S_*y/x*_/a*, respectively, where S_*y/x*_ was the standard deviation of the residuals, and *a* was the slope of the calibration curve. As it is reported in Table 2, LOD and LOQ values obtained were <0.3 and 0.7 mg kg^–1^, respectively.

**TABLE 2:** Main analytical parameters and mineral composition of DP by ICP-OES, expressed in mg per kg on dry matter basis (*n* = 3, mean ± SD).

Mineral	DP (mg k g_dm_^–1^)	Analytical parameters	
		
		LOD (mg kg^–1^)	LOQ (mg kg^–1^)
Al	943 ± 68	0.03	0.10
Ca	7,077 ± 64	0.02	0.06
Co	<LOD	0.03	0.09
Cr	<LOD	0.20	0.60
Cu	64 ± 1	0.01	0.03
Mg	1,851 ± 30	0.06	0.20
Mn	58 ± 1	0.03	0.11
K	1,467 ± 38	0.09	0.30
Fe	562 ± 15	0.04	0.14
Na	628 ± 20	0.03	0.11
Ni	<LOQ	0.03	0.11
Zn	135 ± 1	0.04	0.12
Si	81 ± 10	0.20	0.60
P	819 ± 5	0.010	0.03
S	1,066 ± 8	0.04	0.13

The mineral concentrations found in DP (Table 2) were determined and followed the order: Ca > Mg > K > S > Al > P > Na > Fe. These results agree with those reported by Vejnovic et al. (54) on natural grasslands. Nag et al. (55) analyzed elements and metalloids with natural or anthropogenic origin, and depicted a classification based on their function in biological systems. They classified Ca, Mg, K, P, and S (major elements found in DP) as essential for life processes, such as keeping electrical neutrality in cells and producing actions in muscles and nerves. In this study, Na to K ratio was 0.43, very close to the ratio recommended by WHO/FAO for an adult human (0.49) (56). A high concentration of Ca was found, which is a main structural mineral helping in metabolism, serving as a signal for vital physiological processes.

Regarding Mg, it is the fourth most abundant cation in the body and plays a fundamental role as a cofactor in several enzymatic reactions involved in energy metabolism (56). The Fe content in DP, an important micro-mineral, was higher than results reported by Al-Rowaily et al. (50) on five wild geophytes (*C. capitatus, C. conglomeratus, Elymus farctus, Lasiurus scindicus*, and *Panicum turgidum*). Similar results were obtained by Bado et al. (57) when evaluating three morphotypes of *C. esculentus* tubers with K, P, Si, Cl, S, and Mg as the most abundant minerals. Finally, it is important to point out that K is the main mineral present in *horchata*, followed by P, Mg, and Ca (58). Potassium was also found in a significant concentration in DP, and it has been reported to be one of the main minerals present in dried plants (2, 47, 59). As a result of this work, it can be concluded that DP has a great potential to be used as a rich source of important minerals in nutrition applications.

### Monosaccharides Composition

**FIGURE 2:**
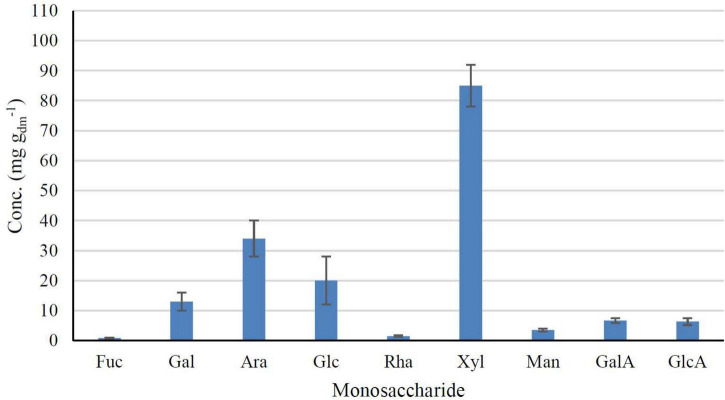
Monosaccharides composition of DP by TFA hydrolysis, expressed as mg per g in a dry matter basis [Glucose (Glc), fucose (Fuc), galactose (Gal), arabinose (Ara), xylose (Xyl), mannose (Man), rhamnose (Rha), galacturonic acid (Gal), and glucuronic acid (GlcA); (*n* = 3, mean ± SD)].

The monosaccharides composition was determined to understand the properties, bioactivity and possible applications of carbohydrates present in DP. Acid hydrolysis is the most used method for hydrolyzing polysaccharide chains. Glycosidic bonds are destroyed during hydrolysis and oxygen atoms are protonated and cleaved, isolating monosaccharides which are usually quantified by HPAEC-PAD (37). Figure 2 shows the carbohydrate composition of DP using TFA hydrolysis. DP was mainly composed of xylose (85 ± 7 mg g_dm_^–1^) and lower amounts of arabinose (34 ± 6 mg g_dm_^–1^), glucose (20 ± 8 mg g_dm_^–1^), and galacturonic acid (13 ± 3 mg g_dm_^–1^). The total carbohydrate content, calculated as the sum of all free sugars, was 171 ± 31 mg g_dm_^–1^, indicating a clear potential of this by-product to be used as a source of carbohydrates. Glucose is an energy source for many organisms, from bacteria to humans, being the brain the most critical site of glucose every day, using 75% (60). Marchyshyn et al. (61) reported that xylose has antifungal and antibacterial properties since its aldopentose can particularly affect Gram-negative organisms and *Candida* species. Xylose can be applied in clinical medical practices since it stimulates the growth of “friendly flora” in the intestines, thus enhancing the production and assimilation of all foods and reinforcing the immune system to help fighting off any disease.

These authors also analyzed the monosaccharides and their derivatives after hydrolysis from tiger nut and *C. esculentus* L. fresh plant. The major monosaccharide found was xylose, but reporting a much lower concentration (39.07 mg g_dm_^–1^) than obtained in this work. In addition, similar contents of arabinose (18.27 mg g_dm_^–1^), glucose (23.54 mg g_dm_^–1^), and galacturonic acid (9.57 mg g_dm_^–1^) were obtained in the fresh plant, in agreement with the results obtained in the present work for DP. Different sugar composition was reported in the literature for rice straw, a similar agricultural by-product, showing values of xylose, glucose and arabinose of 263 ± 11, 33 ± 3, and 34 ± 2 μg mg^–1^, respectively (62).

**FIGURE 3:**
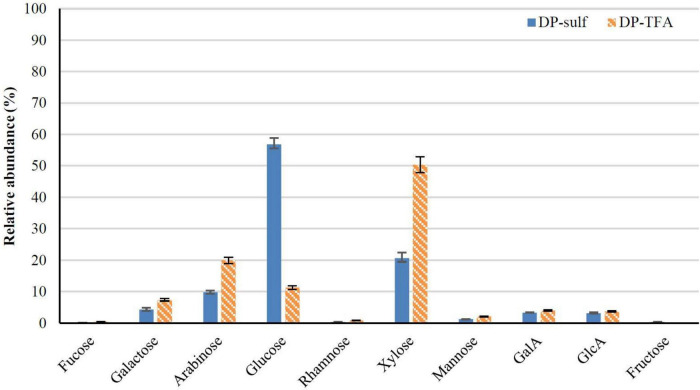
Monosaccharide composition of DP calculated by TFA (*DP-TFA*) and sulfuric acid hydrolysis (*DP-sulf*), expressed as relative abundance (%, g per 100 g of total free sugars) (*n* = 3, mean ± SD).

In order to estimate the crystalline cellulose present in DP, a two-step hydrolysis process was performed. The relative abundances of sugar units determined after TFA hydrolysis and two-step sulfuric hydrolysis are shown in Figure 3. One of the main differences between these two hydrolysis processes is that TFA cannot digest crystalline cellulose. In contrast, the two-stage hydrolysis process facilitates the subsequent breaking of the glycosidic bonds and their separation from insoluble lignin by hydrolysis with sulfuric acid (37, 63, 64). Consequently, a reasonable estimation of the crystalline cellulose content can be obtained by subtracting the glucose value obtained from the TFA hydrolysis from the total glucose, calculated by the sulfuric acid hydrolysis. As a result, approximately 46% of crystalline cellulose was obtained, suggesting that this by-product could be a potential source of micro or nanocellulose and corroborating the results discussed in section “Chemical Composition of Dry Powder” above.

### Microwave-Assisted Extraction Optimization From Dry Powder

Microwave-assisted extraction conditions were optimized using a BBD with a total of 29 runs, considering the effect of four independent variables (temperature, ethanol concentration, extraction time, and solvent volume). Table 3 shows the results obtained for extraction yield, TPC and antioxidant capacity (ABTS), with values ranging from 1.38 to 2.40%, 76.8 to 142.2 mg_*GAE*_ 100 g_dm_^–1^, and 4.04 to 8.12 μmol_trolox_ g_dm_^–1^, respectively, obtaining the highest values for the studied responses for run 22. The experiments corresponding to the central points (runs: 1, 5, 6, 19, and 26) resulted in mean values of 1.87 ± 0.13% (RSD = 7.1%), 106 ± 8 mg_*GAE*_ 100 g_dm_^–1^ (RSD = 7.5%) and 6.0 ± 0.4 μmol_trolox_ g_dm_^–1^ (RSD = 6.6%) for extraction yield, TPC and ABTS, respectively; providing acceptable RSD values and an adequate agreement with the model.

**TABLE 3:** Box–Behnken experimental design matrix and data obtained for the studied responses (yield, TPC, and ABTS) after MAE of DP.

Run	Independent variables				Responses		
		
	X_1_ Temperature (°C)	X_2_ Ethanol (%)	X_3_ Extraction time (min)	X_4_ Extraction volume (mL)	Extraction yield (%)	TPC (mg_*GAE*_ 100 g_dm_^–1^)	ABTS (μ mol_*t*rolox_/g_dm_^–1^)
1	0 (60)	0 (60)	0 (22.5)	0 (65)	1.99	113.69	6.29
2	+1 (80)	0 (60)	+1 (40)	0 (65)	2.20	129.31	6.92
3	−1 (40)	0 (60)	+1 (40)	0 (65)	1.78	95.41	5.47
4	+1 (80)	+1 (80)	0 (22.5)	0 (65)	1.92	100.85	5.43
5	0 (60)	0 (60)	0 (22.5)	0 (65)	1.71	92.58	5.31
6	0 (60)	0 (60)	0 (22.5)	0 (65)	1.99	108.06	6.17
7	+1 (80)	−1 (40)	0 (22.5)	0 (65)	2.36	130.45	7.12
8	+1 (80)	0 (60)	−1 (5)	0 (65)	2.02	113.48	6.48
9	0 (60)	+1 (80)	0 (22.5)	+1 (80)	1.87	95.82	5.79
10	−1 (40)	0 (60)	−1 (5)	0 (65)	1.57	86.50	5.03
11	0 (60)	−1 (40)	+1 (40)	0 (65)	1.76	102.63	5.79
12	+1 (80)	0 (60)	0 (22.5)	−1 (50)	1.60	89.19	5.03
13	0 (60)	+1 (80)	+1 (40)	0 (65)	1.70	82.59	5.09
14	0 (60)	−1 (40)	0 (22.5)	+1 (80)	2.29	128.92	7.72
15	0 (60)	0 (60)	−1 (5)	−1 (50)	1.91	106.24	6.04
16	0 (60)	0 (60)	+1 (40)	+1 (80)	2.35	134.67	7.66
17	0 (60)	0 (60)	−1 (5)	+1 (80)	1.88	109.72	6.37
18	+1 (80)	0 (60)	0 (22.5)	+1 (80)	2.30	131.91	7.60
19	0 (60)	0 (60)	0 (22.5)	0 (65)	1.92	108.81	6.13
20	0 (60)	+1 (80)	0 (22.5)	−1 (50)	1.57	85.20	4.65
21	0 (60)	+1 (80)	−1 (5)	0 (65)	1.59	78.34	4.39
22	0 (60)	−1 (40)	0 (22.5)	−1 (50)	2.40	142.16	8.12
23	−1 (40)	0 (60)	0 (22.5)	+1 (80)	1.88	103.40	6.08
24	0 (60)	−1 (40)	−1 (5)	0 (65)	2.29	127.08	7.38
25	0 (60)	0 (60)	+1 (40)	−1 (50)	1.38	79.70	4.59
26	0 (60)	0 (60)	0 (22.5)	0 (65)	1.75	106.42	5.85
27	−1 (40)	−1 (40)	0 (22.5)	0 (65)	1.57	91.57	4.99
28	−1 (40)	0 (60)	0 (22.5)	−1 (50)	1.74	100.31	5.66
29	−1 (40)	+1 (80)	0 (22.5)	0 (65)	1.55	76.85	4.04

According to the multiple regression analysis, the following quadratic polynomial empirical Eqs 4–6, describing the relation between each response variable and the independent variables, were obtained; where X_1_, X_2_, X_3_, and X_4_ correspond to temperature, ethanol concentration, extraction time and solvent volume, respectively.


$$E\,x\,t\,r\,a\,c\,t\,i\,o\,n\,y\,i\,e\,l\,d=6.7037+0.00093\,X_{1}-0.03594\,X_{2}-0.08451\,X_{3}$$

$$-0.09729\,X_{4}-0.00004\,X_{1}^{2}-0.00026\,X_{1}\,X_{2}-0.00002\,X_{1}\,X_{3}$$

$$+0.00046\,X_{1}\,X_{4}+0.00007\,X_{2}^{2}+0.00045\,X_{2}\,X_{3}+0.00034\,X_{2}\,X_{4}$$

(4) $$-0.00008\,X_{3}^{2}+0.00095\,X_{3}\,X_{4}+0.00029\,X_{4}^{2}$$

$$T\,P\,C=418.201-0.54195\,X_{1}-1.21917\,X_{2}-4.35568\,X_{3}$$

$$-6.80656\,X_{4}-0.00474\,X_{1}^{2}-0.00474\,X_{1}\,X_{2}+0.00494\,X_{1}\,X_{3}$$

$$+0.03302\,X_{1}\,X_{4}-0.00685\,X_{2}^{2}+0.02049\,X_{2}\,X_{3}+0.01988\,X_{2}\,X_{4}$$

(5) $$-0.00765\,X_{3}^{2}+0.04904\,X_{3}\,X_{4}+0.02379\,X_{4}^{2}$$

$$A\,B\,T\,S=26.1677+0.00829\,X_{1}-0.09998\,X_{2}-0.26121\,X_{3}$$

$$-0.04693\,X_{4}-0.00055\,X_{1}^{2}-0.00046\,X_{1}\,X_{2}+0.000004\,X_{1}\,X_{3}$$

$$+0.001786\,X_{1}\,X_{4}-0.000345\,X_{2}^{2}+0.00164\,X_{2}\,X_{3}$$

(6) $$+0.00128\,X_{2}\,X_{4}-0.00016\,X_{3}^{2}+0.00261\,X_{3}\,X_{4}+0.00204\,X_{4}^{2}$$

Analysis of variance was carried out to test the model reliability and to evaluate the influence of the studied variables in the selected responses (Table 4). The obtained results showed coefficients of determination for extraction yield, TPC and ABTS of 0.7959, 0.8061, and 0.8446, respectively, indicating the model’s accuracy and a reasonable degree of correlation between experimental and predicted values (62). In addition, the lack of fit was not significant, obtaining high *p*-values (*p* > 0.05) (0.2186, 0.1868, and 0.1584 for yield, TPC, and ABTS, respectively), verifying the good fit of the proposed models to predict polyphenols extraction from DP under the studied experimental region (65). According to the obtained *F* values, the most and least influencing factors on the studied responses were ethanol concentration and time, respectively. The increment of ethanol concentration significantly influenced (*p* < 0.05) extraction yield, TPC value and antioxidant activity, measured by the ABTS method, with a negative effect. Conversely, temperature and solvent volume showed significant (*p* < 0.05) positive interactions in all the studied responses, whereas time was not significant (*p* > 0.05) in any case.

**TABLE 4:** Analysis of variance obtained for response surface quadratic models in DP extraction.

Source	Degrees of freedom	Extraction yield (%)				TPC (mg GAE/100 g_dm_)				ABTS (μ mol_trolox_/g_dm_)			
				
		Sum of squares	Mean square	*F*-value	*P*-value	Sum of squares	Mean square	*F*-value	*p*-Value	Sum of squares	Mean square	*F*-value	*P*-value
X_1_: temperature (°C)	1	0.44	0.44	25.33	0.0073*^a^*	1,660.11	1,660.11	26.42	0.0068*^a^*	4.46	4.46	29.08	0.0057*^a^*
X_2_: ethanol concentration (%v/v)	1	0.51	0.51	29.11	0.0057*^a^*	3,439.18	3,439.18	54.73	0.0018*^a^*	11.44	11.44	74.69	0.0010*^a^*
X_3_: time (min)	1	0.00	0.00	0.04	0.8523	0.72	0.72	0.01	0.9199	0.00	0.00	0.01	0.9153
X_4_: extraction volume (mL)	1	0.32	0.32	18.54	0.0126*^a^*	860.91	860.91	13.70	0.0208*^a^*	4.23	4.23	27.58	0.0063*^a^*
X_1_^2^	1	0.00	0.00	0.12	0.7466	23.39	23.39	0.37	0.5748	0.32	0.32	2.07	0.2240
X_1_X_2_	1	0.04	0.04	2.47	0.1909	55.34	55.34	0.88	0.4012	0.14	0.14	0.89	0.3978
X_1_X_3_	1	0.00	0.00	0.02	0.8951	11.98	11.98	0.19	0.6849	0.00	0.00	0.00	0.9948
X_1_X_4_	1	0.08	0.08	4.46	0.1022	392.53	392.53	6.25	0.0668	1.15	1.15	7.50	0.0520
X_2_^2^	1	0.01	0.01	0.30	0.6121	48.80	48.80	0.78	0.4280	0.12	0.12	0.81	0.4196
X_2_X_3_	1	0.10	0.10	5.82	0.0733	205.81	205.81	3.28	0.1446	1.32	1.32	8.62	0.0425*^a^*
X_2_X_4_	1	0.04	0.04	2.46	0.1921	142.32	142.32	2.26	0.2068	0.59	0.59	3.87	0.1206
X_3_^2^	1	0.01	0.01	0.27	0.6305	35.62	35.62	0.57	0.4934	0.02	0.02	0.11	0.7539
X_3_X_4_	1	0.25	0.25	14.50	0.0190*^a^*	662.87	662.87	10.55	0.0314*^a^*	1.87	1.87	12.21	0.0250*^a^*
X_4_^2^	1	0.03	0.03	1.61	0.2731	185.96	185.96	2.96	0.1605	1.38	1.38	8.98	0.0401*^a^*
Lack-of-fit	10	0.40	0.04	2.30	0.2186	1,623.33	162.33	2.58	0.1868	4.44	0.44	2.90	0.1584
Pure error	4	0.07	0.02			251.36	62.84			0.61	0.15		
Cor total	28	2.31				9,666.18				32.51			
*R* ^2^		0.7959				0.8061				0.8446			

*^a^Significant at p < 0.05.*

**FIGURE 4:**
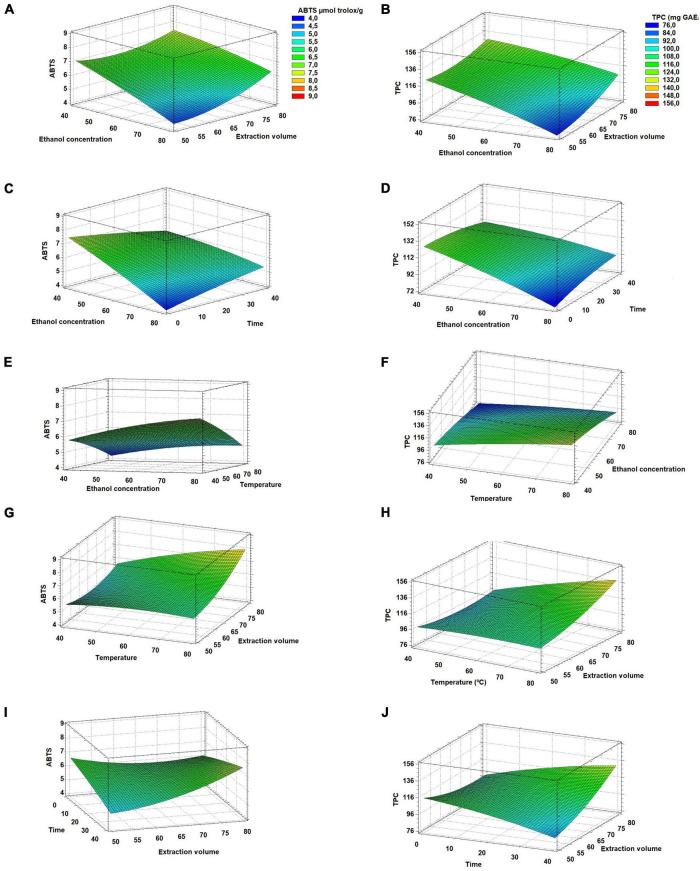
**(A–J)** Response surface plots showing significant interactions between independent variables on ABTS (left column) and TPC (right column).

These interactions between extraction variables were studied through 3D response surface plots to show graphically their influence on maximizing polyphenols content and antioxidant activity. Figure 4 shows the obtained response surfaces for TPC and ABTS by plotting the effects of two variables at a different level, from −1 to +1, and keeping the other variables at their center point (0). A significant positive interaction was observed between time and extraction volume (Figures 4I,J), enhancing TPC and antioxidant activity. The highest ABTS and TPC values were obtained when time and ethanol concentration were fixed, and extraction temperature and solvent volume were varied (Figures 4G,H). At high solvent-sample ratio values, elevated temperatures should be used in order to maximize the responses. High microwave power and long times would favor the extraction of polyphenols without degradation, resulting in higher values for the response variables (66). Although the increase in temperature of the overall extraction process could produce the degradation and partial loss of volatile compounds, it can also improve the interaction and contact of the analytes with the solvent (67). Besides, it is widely known that an increment in extraction volume can increase the dissolution strength of polyphenolic compounds from plants, facilitating their diffusion into the solvent (68, 69).

The negative influence of ethanol concentration was also observed in the response surfaces (Figures 4A–F), showing higher response values when approaching low ethanol concentrations. These surfaces showed the steepest slopes compared to the rest, as ethanol concentration was the variable showing the highest significance (*p* < 0.05). According to Yang et al. (70), water and ethanol at low concentrations can easily reach plant cells. Similarly, it is well known that ethanol is a low polar solvent while water is a strong one, and both can be easily mixed at different ratios, increasing considerably the solvent polarity and making it suitable for the extraction of polar compounds, such as polyphenols (38). However, a high concentration of ethanol can negatively affect the efficient extraction of polyphenols due to interferences in the extraction produced by the protein’s denaturation. Besides, high amounts of ethanol could dehydrate the vegetable cells, hindering the polyphenols diffusion from the plant matrix to the solvent, resulting in reduced extraction yields. Feki et al. (71) used MAE to extract polyphenols from Jojoba (*Simmondsia chinensis*) seeds and they found that a concentration of around 36% of ethanol in water enabled the effective extraction of polyphenols, including polar, weak-polar and non-polar compounds. Consequently, the MAE method has proven to be a valid technique to obtain good yields. The optimal conditions should be validated to verify that the experimental results fit to the statistical design used in this study.

#### Validation of Optimum Extraction Conditions

Desirability functions are used to search for a combination of the different variable levels that simultaneously satisfy all the requirements for each response variable (72). In this study, a desirability value of 1 was found when the experimental conditions were: 80°C, 40% (v/v) ethanol, 18 min and 77 mL of solvent. Verification experiments under these optimal conditions were carried out in triplicate. The obtained experimental results (Table 5) were quite close to those predicted by the polynomial quadratic models (Eqs 4–6), so it can be concluded that the optimized MAE method was validated for polyphenols extraction from DP within the range of independent variables studied.

**TABLE 5:** Optimal conditions for the extraction of active compounds in DP and response variables evaluated (*n* = 3, mean ± SD).

	Independent variable				
	
	Temperature (°C)	Ethanol concentration (% v/v)	Time (min)	Extraction volume (mL)	
**Optimal conditions**	80	40	18	77	

**Responses**	**Extraction yield (%)**	**TPC (mg _*GAE*_ 100 g_dm_^–1^)**	**ABTS (μ mol_*t*rolox_ g_dm_^–1^)**	**FRAP (μ mol_*t*rolox_ g_dm_^–1^)**	**EC_50_ DPPH (mg mL^–1^)**

Experimental results	2.27 ± 0.09	136.00 ± 3.00	8.41 ± 0.09	13.40 ± 0.60	32.80 ± 0.90
Predicted results	2.52	142.88	8.13	**–**	**–**

The overall extraction yield was 2.27 ± 0.09% which can be considered a good result, compared with those reported by other authors. Jing et al. (32) studied different extraction methods (traditional reflux, UAE, MAE, UAE followed by MAE, and DHPM) to obtain flavonoids from fresh leaves of *C. esculentus* L. by using ethanol:water mixtures. Results obtained regarding flavonoid yield by using these methods ranged from 0.64 to 1.46%. The highest yield was obtained by using DHPM, with a pressure of 120 MPa, a percentage of ethanol of 60%, an extraction temperature of 80°C and an extraction time of 90 min. However, the yield obtained by MAE, using 60% (v/v) ethanol in water, a dry matter:solvent ratio of 1:16, 120 s of extraction time and a microwave power of 30%, was 1.34%, much lower than the result obtained in the present work. In another study, the applicability of microwave irradiation to assist the extraction of essential oils from *C. rotundus* achieved a yield of 1.24% (73).

In different studies with plants, the concentration of polyphenols was reported to be dependent on the harvesting practices and the moisture degree of the plant (18, 74, 75). Despite the importance of this subject, there is limited literature based on MAE from the *C. esculentus* L., plant, with most of the articles published on this matter being based on tiger nut by-product extraction and the interaction between its phenolic profile and antioxidant properties (5, 26, 42).

Regarding TPC, a value of 136 ± 3 mg_*GAE*_ 100 g_dm_^–1^ was obtained at the optimal MAE conditions. Compared to other perennial plants, such as rice straw, which is also cultivated and widespread in a similar way to tiger nut in the same area, Karimi et al. (76) found TPC values of 2.11 ± 0.20, 3.18 ± 0.14 and 2.56 ± 0.22 mg_*GAE*_ g_dm_^–1^ for straw methanolic extracts of *Ali Kazemi*, *Hashemi*, and *Khazar* rice varieties, respectively. In another work, Jablonsky et al. (77) evaluated TPC values of various spruce bark extracts obtained using different deep eutectic solvents (DESs), ranging from 233.6 to 596.2 mg_*GAE*_ 100 g_dm_^–1^. It is important to highlight that polyphenolic compounds have proven antioxidant and antimicrobial capacities and that by incorporating them into our diet, they may prevent/limit nutritional disorders or important diseases such as brain disorders or cancer (78–81). Al-Rowaily et al. (49) focused their study on five wild geophytes (plants with underground storage organs that include bulbs, corms, tubers, and rhizomes), such as *C. capitatus, C. conglomeratus, E. farctus, L. scindicus*, and *P. turgidum*, with a high diversity in Mediterranean-type ecosystems. A variable content of total polyphenols (16.02 ± 0.86, 26.34 ± 1.41, 13.57 ± 0.73, 9.59 ± 0.51, and 10.69 ± 0.57 mg g_dm_^–1^, respectively) were found in these plants. Tomé and da Silva (82) evaluated the antioxidant capacity in aqueous, hydroethanolic and ethanolic extracts of basil, parsley, rosemary, thyme, chervil and chives by the ABTS method. The obtained results ranged from 9.13 to 40.44 μmol_*t*rolox_ g_dm_^–1^.

The antioxidant capacity of MAE extracts was also evaluated by using two additional colorimetric assays, such as DPPH and FRAP, which are based on different reaction mechanisms, radical and complex formation, respectively (83). Table 5 shows the results obtained for these tests. These results demonstrated the potential of DP extracts to be used as antioxidant sources and they were comparable with other studies using similar plants or by-products. In a work developed by Surveswaran et al. (84), the antioxidant capacity of 133 Indian medicinal plant species sampled from 64 families was evaluated by the ABTS and FRAP assays, with values ranging from 0.16 to 500.70 mmol_*t*rolox_ 100 g_dm_^–1^ and 0.16 to 124.05 μmol_*t*rolox_ g_dm_^–1^, respectively. *Cyperus articulatus* extracts, belonging to the same Cyperaceae family as DP, also showed antioxidant activity (DPPH EC_50_ of 16.9 ± 0.1 μg mL^–1^, ABTS of 2.28 ± 0.08 mmol_trolox_ g_extract_^–1^, FRAP of 13.4 ± 0.6 μmol_*t*rolox_ g_dm_^–1^). A methanolic extract obtained from the *C. rotundus* L. root, also belonging to the Cyperaceae family, showed lower antioxidant capacity, with a FRAP value of 1.74 μmol_*t*rolox_ g_dm_^–1^ (85). On the other hand, Datta et al. (86) evaluated different extracts from *Cyperus compressus*, where the 70% ethanol extract showed a FRAP value of 2.196 ± 0.002 μmol_*t*rolox_ g_dm_^–1^, lower than results obtained in this study. Similarly, another study evaluated the antioxidant capacity by FRAP of 13 plant extracts (87) obtained by dilution with a mixture of acetone/ultrapure water/glacial acetic acid 70:28:2% (v/v) using an Ultra-Turrax stirrer. In this case, the results obtained for extracts from fresh plants ranged from 43.61 to 472.32 μmol_*t*rolox_ g_dm_^–1^.

Finally, the value obtained for the scavenging activity of the DPPH radical (EC_50_) for the extract obtained under the optimal conditions was 32.80 mg mL^–1^. Results obtained in this work were higher compared to those found for the wild edible plant *Raphanus raphanistrum L.* (3.12 ± 0.07 mg mL^–1^ in hydroalcoholic extracts) (40). Karimi et al. (76) obtained lower EC_50_ values (258.7 ± 2.65, 218.5 ± 3.01 and 248 ± 1.87 μg mL^–1^) for the *Ali Kazemi*, *Hashemi*, and *Khazar* varieties, respectively. Jing et al. (32) obtained lower results for the EC_50_ value using various extraction techniques from the fresh plant of *C. esculentus* L. ranging from 0.17 to 0.48 mg mL^–1^. Siroua et al. (85) evaluated the EC_50_ value by the DPPH method of the hydroalcoholic extract (ethanol/water 80:20, v/v) of *C. rotundus* rhizomes, obtaining values of 46.12 ± 0.55 mg mL^–1^. They also evaluated the effect of dilution with different fractions of hexane, chloroform, ethyl acetate, butanol, or methanol, obtaining EC_50_ results ranging from 0.75 to 50.78 mg mL^–1^.

In conclusion, the obtained results demonstrated the potential of DP extracts to be used as antioxidant agents in nutritional, food, cosmetics and packaging applications, according to the TPC, ABTS, FRAP, and DPPH results; which are reported in the present work for the first time being comparable with results published for other similar plants or subspecies of the same family.

### Phenolic Profile of Dry Powder Extract

**FIGURE 5:**
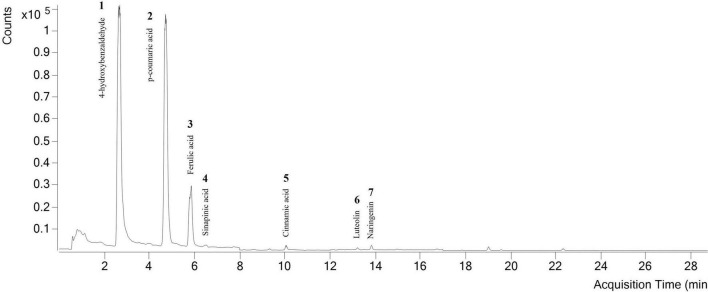
Chromatogram obtained by UHPLC-ESI-MS/MS from the DP extract peak assignations.

Table 6 and Figure 5 show the main compounds identified by UHPLC-ESI-MS/MS in the DP extract obtained under optimal conditions by MAE. The chromatographic method was optimized, operating under both, negative and positive modes, to find the best fragmentation pattern for each compound. Quantification was performed using multiple reaction monitoring mass spectrometry (MRM-MS). All calibration curves showed acceptable linearity levels with coefficients of determination (*R*^2^) ranging from 0.9910 to 0.9992 for the studied analytes, at seven calibration points (concentration range 0.1–15.0 mg kg^–1^), in triplicate. 4-Hydroxybenzaldehyde, ferulic acid, sinapinic acid, cinnamic acid, luteolin, and naringenin were obtained by positive ionization and the detection of [M+H]^+^ molecular ions. Among them, the highest concentration was obtained for ferulic acid, 4.07 ± 0.01 mg 100 g_dm_^–1^, followed by 4-hydroxybenzaldehyde, cinnamic acid, luteolin, naringenin, and sinapinic acid. In addition, *p*-coumaric acid was determined by negative ionization, with a concentration of 7.67 ± 0.16 mg 100 g_dm_^–1^, resulting in the major polyphenol present in the DP extract.

**TABLE 6:** Analytical parameters and concentration of main polyphenols identified in the DP extract under optimal MAE conditions (*n* = 3, mean ± SD).

Compound*	Rt (min)	Linearity (*R*^2^)	Ionization mode	Precursor ion	Quantitation ion (m/z)	Identification ions (m/z)	Concentration (mg 100 g_dm_^–1^)
4-Hydroxybenzaldehyde (1)	2.6	0.9992	**+**	[M+H]^+^	123.2	95.2, 77.2, 51.1	1.28 ± 0.06
p-Coumaric acid (2)	4.7	0.9959	−	[M−H]^–^	163.1	145.2, 119.2, 103.1	7.67 ± 0.16
Ferulic acid (3)	5.9	0.9975	+	[M+H]^+^	195.2	134.3, 106.0, 89.2	4.07 ± 0.01
Sinapinic acid (4)	6.5	0.9981	+	[M+H]^+^	225.4	206.8, 175.3, 119.3	0.50 ± 0.01
Cinnamic acid (5)	10.1	0.9969	+	[M+H]^+^	149.2	131.3, 103.2, 77.2	1.10 ± 0.03
Luteolin (6)	13.2	0.9910	+	[M+H]^+^	287.2	269.0, 240.9, 153.3	1.03 ± 0.01
Naringenin (7)	13.8	0.9938	+	[M+H]^+^	273.3	153.3, 147.3, 119.2	0.60 ± 0.01

*The number refers to the peaks shown in the chromatogram of Figure 5.

The obtained phenolic composition was related to the antioxidant performance shown for the extract, indicating that these compounds play a key role in such functionality. To the best of our knowledge, this is the first work reporting the identification and quantification of phenolic compounds present in DP. Taheri et al. (44) described the antioxidant capacity of *Cyperus* spp., concluding that it can be attributed to a plethora of phytochemicals present in its composition. Jing et al. (32) obtained a flavonoid extract with antioxidant and antimicrobial properties from *C. esculentus*, but no polyphenols profile was reported. The HPLC analysis of *C. rotundus*, another plant from the Cyperaceae family, showed the presence of six phenolic compounds, namely gallic acid, coumaric acid, kaempferol, naringenin, rutin, and quercetin (77). Datta et al. (86) performed the HPLC analysis of a 70% aqueous ethanol extract obtained from another plant of the family, in this case *C. compressus*, to identify the polyphenolic compounds and flavonoids present. Vanillic acid, ferulic acid, rutin, myricetin, quercetin, and apigenin were detected and quantified. Ferulic acid appeared in lower concentrations than those obtained in this study (0.218 ± 0.001 mg 100 g_dm_^–1^). *p*-Coumaric acid was also detected in extracts from *C. rotundus* rhizomes (85, 88). The presence of vanillin and *p*-coumaric acid derivatives can reduce the risk of developing several disorders, such as cancer and cardiovascular diseases. And they have shown good bioavailability and bio-accessibility (89). In addition, the presence of ferulic acid as a natural antioxidant is widely known to be directly bound to the plant cell wall cross-linking arabinoxylans (90). Guo et al. also reported the presence and interactions of proteins and anthocyanins in grape skin extracts (91).

Several works have studied the identification and quantification of polyphenols in tiger nuts (23, 27, 92), reporting the presence of most of the phenolic compounds identified in this study. These results are quite similar to those reported by Parker et al. (93) on peeled tiger nuts and their skin extracted with 0.1 M NaOH for 1 h. These authors identified some monomeric phenols, such as vanillin, vanillic acid, p-hydroxybenzoic acid, p-hydroxybenzaldehyde, p-trans-coumaric acid, p-cis-coumaric acid, trans-ferulic acid, and cis-ferulic acid in higher amounts than those obtained in the DP extract. The concentrations of *p*-coumaric acid, ferulic acid and 4-hydroxybenzaldehyde in tiger nut skin were 617, 291, and 337 μg g^–1^, respectively. Moreover, authors indicated that the continuous degradation of the lignin structure is a key factor influencing phenolics extraction and content in the final extracts, particularly for *p*-coumaric and ferulic acids. Oladele et al. (94) also studied the polyphenols profile of different types of tiger nuts and they found high concentrations of ferulic acid (34–58 mg 100 g_dm_^–1^), p-hydroxybenzaldehyde (≈ 16 mg 100 g_dm_^–1^), *p*-coumaric acid (≈ 17 mg 100 g_dm_^–1^), and sinapinic acid (≈ 21 mg 100 g_dm_^–1^). Ezeh et al. (95) also detected high polyphenols content in mechanically pressed tiger nut oil with antioxidant activity, protecting the oil from oxidative rancidity and prolonging its shelf-life. Soto Mayer (96) reported that the compounds in higher concentrations in methanolic tiger nut extracts after treatment in an ultrasonic water bath were chlorogenic acid, ferulic acid, and luteolin. Jahan et al. (97) found concentrations for gallic acid (6.80 ± 0.13 mg 100 g_dm_^–1^), chlorogenic acid (2.91 ± 0.07 mg 100 g_dm_^–1^), *p*-coumaric acid (11.67 ± 0.12 mg 100 g_dm_^–1^) and ferulic acid (4.12 ± 0.06 mg 100 g_dm_^–1^) similar to those obtained in the present study in the rhizomes of another plant of the tiger nut family (*C. rotundus*).

## Conclusion

In the present study, the complete characterization of the harvesting residues generated from the *C. esculentus* L. plant used in the production of the *horchata* beverage was carried out to demonstrate the presence of valuable compounds from the nutritional point of view with potential use by the food industry. In addition, innovative extraction strategies have been applied for such purpose, resulting in the valorization of these residues and enhancement of the circular economy by using good agricultural practices and waste management in specific areas of the Mediterranean basin where tiger nut is produced. The DP has shown potential for high value applications, especially by its important antioxidant performance and the large number of major polyphenols present in its composition. Optimal MAE conditions to extract polyphenolic compounds from DP were determined by using RSM; being the optimal conditions 80°C, 18 min, ethanol proportion of 40% (v/v), and solvent volume of 77 mL. These conditions provided the maximum extraction yield (2.27 ± 0.09%), a TPC value of 136 ± 3 mg_*GAE*_ 100 g_dm_^–1^ and an antioxidant capacity by the ABTS method of 8.41 ± 0.09 μmol_*trolox*_ g_dm_^–1^. Antioxidant performance was also confirmed by FRAP and DPPH (EC_50_) assays. The main polyphenols present in DP extracts obtained at the optimal conditions were quantified by HPLC-ESI-MS/MS, with *p*-coumaric acid (7.67 ± 0.16 mg 100 g_dm_^–1^), as the major compound. Therefore, the chemical composition, and in particular, the presence of polyphenols and their antioxidant activity, demonstrated the applicability of this residue as a source of bioactive compounds to be applied as natural antioxidants or preservatives in food applications or by the cosmetic sector. The fortification of foods with extracts obtained from *C. esculentus* L. could be an interesting application to get functional foods.

Moreover, as a source of α-cellulose (47.2 ± 1.8%), this residue can be also considered as the precursor of biopolymers with application by the food packaging sector. New procedures to transform cellulose at a nanometric scale with different morphologies are currently under evaluation and the high content shown by this agricultural residue brings added value and could contribute to the reduction of a severe environmental problem. This work has revealed the presence of relevant nutrients and bioactive molecules in this agricultural residue, such as minerals, proteins, and free sugars, which offer a wide variety of new environmentally friendly applications, helping to limit and solve industrial, economic and environmental problems.

## Data Availability Statement

The raw data supporting the conclusions of this article will be made available by the authors, without undue reservation.

## Author Contributions

CP designed and conducted the experiments, analyzed the data, and wrote the manuscript. MR writing—review and editing. AJ and MG revised the manuscript. All authors participated in conceiving the experiments and interpreting the data and contributed to the article and approved the submitted version.

## Conflict of Interest

The authors declare that the research was conducted in the absence of any commercial or financial relationships that could be construed as a potential conflict of interest.

## Publisher’s Note

All claims expressed in this article are solely those of the authors and do not necessarily represent those of their affiliated organizations, or those of the publisher, the editors and the reviewers. Any product that may be evaluated in this article, or claim that may be made by its manufacturer, is not guaranteed or endorsed by the publisher.
